# Protons, Photons, and the Prostate – Is There Emerging Evidence in the Ongoing Discussion on Particle Therapy for the Treatment of Prostate Cancer?

**DOI:** 10.3389/fonc.2016.00008

**Published:** 2016-01-28

**Authors:** Kilian C. Schiller, Gregor Habl, Stephanie E. Combs

**Affiliations:** ^1^Department of Radiation Oncology, Klinikum rechts der Isar, Technische Universität München (TUM), München, Germany; ^2^Institute of Innovative Radiotherapy (iRT), Department of Radiation Sciences (DRS), Helmholtz Zentrum München, Oberschleißheim, Germany; ^3^Deutsches Konsortium für Translationale Krebsforschung (dktk), Partner Site München, München, Germany

**Keywords:** protons, prostate cancer, carbon ions, clinical trials, IMRT

## Abstract

Proton therapy is actively and repeatedly discussed within the framework of particle therapy for the treatment of prostate cancer (PC). The argument in favor of treating the prostate with protons is partly financial: given that small volumes are treated, treatment times are low, resulting in a hypothetical high patient throughput. However, such considerations should not form the basis of medical decision-making. There are also physical and biological arguments which further support the use of particle therapy for PC. The only relevant randomized data currently available is the study by Zietman and colleagues, comparing a high to a low proton boost, resulting in a significant increase in PSA-free survival in the experimental (high dose) arm (1). With modern photon treatments and image-guided radiotherapy (IGRT), equally high doses can be applied with photons and, thus, a randomized trial comparing high-end photons to protons is warranted. For high-linear energy transfer (LET) particles, such as carbon ions, the increase in relative biological effectiveness could potentially convert into an improvement in outcome. Additionally, through the physical differences of protons and carbon ions, the steeper dose gradient with carbon ions and the lack of beam broadening in the carbon beam lead to a superior dose distribution supporting the idea of hypofractionation. Biological and clinical data are emerging, however, has practice-changing evidence already arrived?

## Introduction

Proton beam therapy (PBT) among particle therapy for prostate cancer (PC) remains a highly topical subject in the uro-oncological community. It is fueled by discussions concerning questionable superiority to photon treatment with regard to survival or local control, higher costs and cost-effectiveness, better tolerance for patients due to fewer side effects, and, last but not least, continuous patient inquiries regarding the therapy (2). This is represented by numerous ongoing trials and publications; the search for “proton therapy AND PC” on clinicaltrials.gov generates 36 hits alone (3). As biological and clinical data are emerging, the question remains: has practice-changing evidence already been uncovered?

## Photons

The goal in radiotherapy (RT) of localized PC is a lethal dose to tumor cells, while ensuring that the smallest possible dose is applied to surrounding tissues, such as rectum and bladder, and thereby avoiding side effects and toxicities for patients.

Nowadays photons are the most commonly used treatment in RT for PC. Photons have no mass and no charge and, therefore, travel easily through target materials. There is an initial increase of energy as they interact with the target material electrons (e.g., the body), which enhances the radiation effect. As a result of this, their peak dose is reached within a few centimeters from the entrance surface – the so-called “dose accumulation effect.” In the deeper trajectory through the body subsequently, the radiation dose decreases until it exits the body. 3D plans initially had a significant dose deposition in the entry and exit fields. With multiple field plans, rapid arc or helical techniques, these doses tend to be significantly smaller, but often a dose bath with low-to-moderate doses over surrounding organs cannot be avoided in order to deliver a deathly dose to cancer cells (4). The possible side effects include gastrointestinal (GI) and genitourinary (GU) problems and a potentially slightly higher risk for secondary malignancies (5). Therefore, photon radiation therapy does not seem appropriate in terms of its physical characteristics to treat those organs located at a great depth within the body. Despite modern improvements in technologies, such as multi-leaf collimators, intensity-modulated radiotherapy (IMRT), or image-guided radiotherapy (IGRT), photon-beam therapy will always include a certain level of entrance and exit doses, resulting in healthy tissue receiving low-to-moderate radiation doses. While these doses are most likely not associated with a prominent side effect risk, such issues necessitate a serious consideration of alternative treatment options, including particle therapy.

## Particle Therapy

As of 2013, more than 123,000 patients had received therapy with heavy particles worldwide, PBT accounting for the majority of this, with over 106,000 patients treated (6). While protons can be termed particles, they are not considered “heavy,” and from their effect they can be categorized as low-linear energy transfer (LET) radiation, comparable to photons. Heavy particles include carbon ions, oxygen as well as neutrons, and others. Particles may be charged (protons, carbon ions) or neutral (neutrons). The term “heavy particle therapy” is generally used to distinguish it from conventional X-Ray RT, which uses massless photons. As most research undertaken so far has investigated protons, we shall mainly focus on them in the following article. Experience with other heavy particles is limited to a mere seven operating carbon ion facilities worldwide, treatment with carbon ions can be considered experimental and, therefore, reliable evidence is only just emerging and no conclusions can yet be drawn with regard to their effectiveness or toxicity.

## Protons

**Figure 1:**
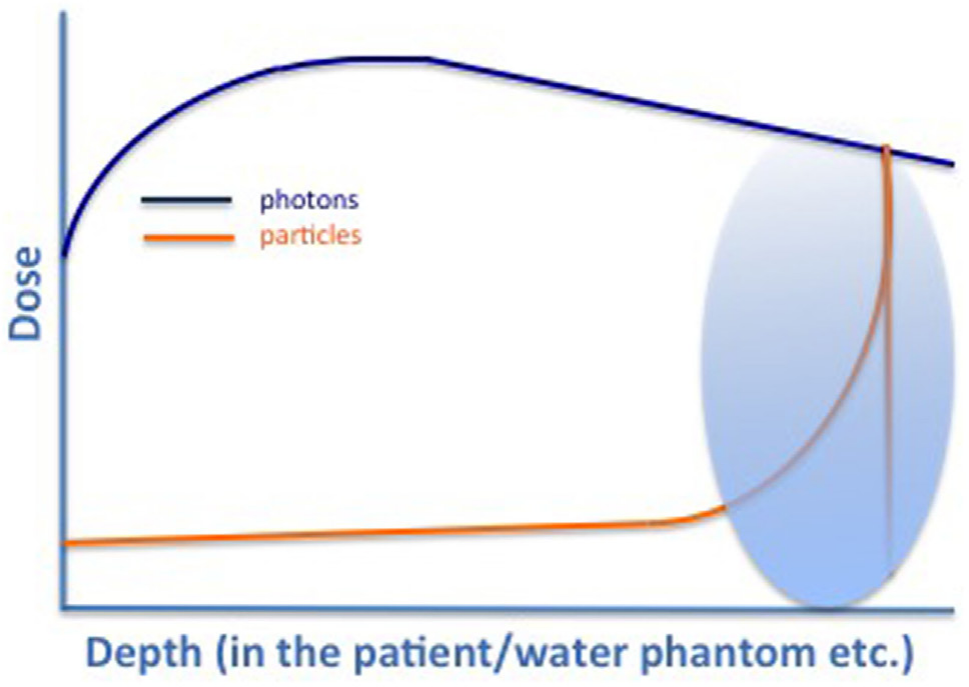
**Characteristic dose profile for photons (green), protons (yellow), and carbon ions (black)**. Typical Bragg Peak (red) for particles that can be directed into defined regions depending on the energy used [adapted from Combs et al. (7)].

Due to their physical characteristics, protons potentially offer a treatment method in which smaller areas receive radiation doses and, thus, bring about fewer side effects. Proton beams are created by a cyclotron or synchrotron, whereby the proton is separated from hydrogen molecules. Protons travel fast through tissue, with minimal room for interaction; in depth the velocity is reduced, interactions occur, and the energy is deposited: they, therefore, stop very abruptly in tissues reaching a very specific depth: the so-called “Bragg peak” (7), see Figure 1. Here, the majority of energy is being deposited. Heavy particles, compared to photons, have a greater radiobiological effect (1.1 times for protons and 2–3 for carbon ions) and, therefore, greater potential to damage cancer cells by interacting more densely with tissue, causing higher levels of ionization per unit length (8, 9). The dose then rapidly decreases to 0 as heavy particles (opposed to photons) stop within the body. Thus, the integral dose with protons is approximately 60% lower than that of any external beam photon technique (10, 11).

In theory, this is ideal for treating tumors near to sensitive structures, such as the brain and spinal cord, as well as for childhood cancers in order to reduce the dose to healthy surrounding organs. PBT can potentially show a clinical superiority compared to photons and is, to a certain extent, established in some populations such as pediatric indications or uveal melanoma, at least in some regions worldwide (12–14).

It is important in all populations to note that RT comes with the risk of developing secondary malignancies years to decades later. Although this risk is low with modern techniques, it is in focus for radiation oncologists, and patients alike. With the sharp dose deposition of particles, there are presumably a smaller number of secondary malignancies due to the lower dose to surrounding healthy structures. Nevertheless PBT, as opposed to photon therapy, generates neutrons as a by-product, which can be scattered into adjacent normal tissues, especially with passive beam techniques. These neutrons have a strong biological effect and, thus, could theoretically increase the risk of secondary malignancies (15). Despite such theoretical concerns, a large retrospective study did not find statistically significant differences in secondary malignancies between patients treated with protons and those treated with photons – 5.2% of all PBT-treated patients had secondary malignancies versus 7.5% in the photon treatment group (16).

The width of the Bragg peak is within the millimeter range and usually not wide enough to cover a whole treatment volume. Several of those peaks must be superimposed to treat effectively a tumor of a certain length and volume – the so-called “spread-out Bragg peak” (SOBP). There can be some uncertainty about the exact location of the Bragg peak due to tissue inhomogeneities. Here, PBT skeptics argue that there is no widely used method for confirming the proton range, or that the SOBP encompasses the prostate *in vivo*, making sufficient margins essential for a successful therapy and thereby diminishing the possible advantage of smaller radiation volumes.

Another technical challenge is that, because of the steep dose gradient (Bragg peak), the plan parameters and patient positioning must be highly precise in order to obtain a high dose within the tumor region while maximizing the protection of organs at risk (OAR). This makes the uncertainty regarding the range of motion in human tissue one of the major hurdles of RT with protons, meaning that particle therapy is more vulnerable to target motion than photon irradiation (17).

Yoon et al. also describe an increased sensitivity to target motion of PBT because of deep dose depletion beyond the SOBP (18).

In RT of PC, the range of motion can be divided into inter- and intrafractional movements. Interfractional movements occur between two radiation appointments, e.g., due to filling of the bladder and rectum. Intrafractional movements happen within one radiation session, e.g., due to breathing, bowel gas, or small patient movements. Because of this, measurements in PBT should be made even during dose delivery and can be accomplished via positron emitters (PET camera) or induced gamma radiation (Compton camera) (19, 20). Motion mitigation strategies are essentially important to exploit the full potential of particle therapy; this includes scanning approaches, such as rescanning, gating, or implementation of motion-surrogates, such as markers (21–23).

## Carbon Ions

As opposed to protons, which can be considered to have radiobiological features similar to photons [relative biological effectiveness (RBE) = 1.1 for protons], other heavy particles have higher LET characteristics, leading to substantial differences in radiobiological interactions. Due to their mass, carbon ions have a higher biological effectiveness compared to protons with a comparable depth-dose profile. The RBE of the carbon ion beams has been estimated as 2.0–3.0 (9). This means that they are twice to three times more effective in killing cancer cells than proton or photon beams as they are more likely to cause deathly DNA double-strand breaks. Thus, carbon ions have radiobiological advantages, including more effective killing of intrinsic radio-resistant tumors, hypoxic tumor cells, and tumor cells in the G0 or S phase (24).

Furthermore, they possess an even sharper dose distribution than protons, but the dose in the region beyond the distal end of the peak is higher in carbon ion beams than proton beams, because carbon ions undergo nuclear interactions producing a fragmentation tail beyond the dose peak (25, 26).

In terms of its medical application, carbon ion therapy has been described as being advantageous *inter alia* for PC. Regarding the anti-tumor effect of carbon ion RT for PC, Ishikawa et al. reported survival data and biochemical relapse-free rates for almost 1000 patients with 20 or 16 fractions at the ion beam center in Chiba, Japan. The 5-year overall survival and cause-specific survival rates for all patients were 95.3 and 98.8%, respectively; the 5-year relapse-free and local control rates were 90.6 and 98.3%, respectively. Especially noteworthy is that the outcomes for biochemical relapse-free survival also included the high-risk group and that hypo-fractionated carbon ion RT seems to have had radiobiological benefit for PC (24, 27). In a more recent Phase I/II study from 2014, Nomiya et al. described a shortened course with only 12 fractions as another feasible option, however, the long-term outcome of such an approach is still pending (28).

**Figure 2:**
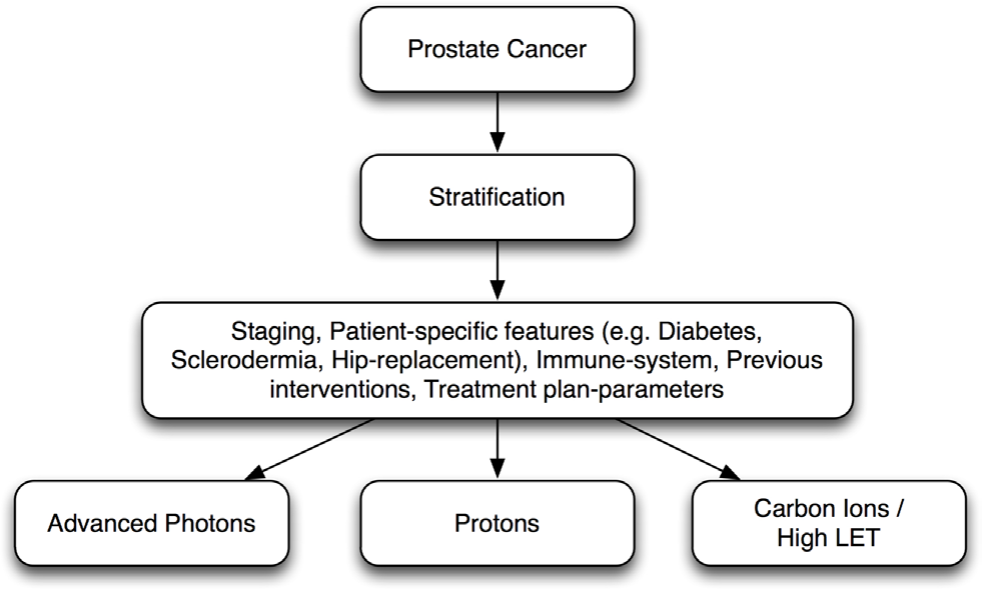
**Individualized radiotherapy (iRT): prostate cancer patient stratification for different radiation modalities**.

Certainly, there are advantages of carbon ion RT, as an option with high biological effectiveness over low-LET radiations, such as photon therapy or PBT, and carbon ion therapy may prove to make a substantial difference in certain patient populations. However, precise definition of clinical study protocols and critical evaluation of patient data together with intake of molecular characteristics of tumors and normal tissue can help to optimally stratify patients for different radiation modalities leading to individualized radiotherapy (*i*RT). (Figure 2).

## Technological Aspects

Common passive scattering systems will be replaced by spot scanning systems in charged particle beam therapy in medium term. It is in routine clinical use at the Paul Scherrer Institute (PSI) in Darmstadt. Advantages are the precise dose distribution and planning options for using intensity-modulated proton beam therapy (IMPT). The application is safe but several aspects for uncertainties, such as the robustness to movements of the target and to IMPT plans, must be taken into account when working with spot scanning techniques. Zhu et al. reported on the single-field integrated boost (SFIB) technique for spot scanning proton therapy based on single-field optimization (SFO) treatment planning techniques (29).

Regarding beam delivery, most experience has mainly been acquired with horizontal beam lines. The success of radiotherapy treatment is strongly increased through the possibility of applying the beam to the target using different angles, such as in IMRT. Hence, the worldwide first gantry for charged particle was brought online in October 2012 at the Heidelberg Ion Therapy Center (HIT), Heidelberg.

## Discussion

The success of irradiation in patients with localized PC correlates with the administered dose, meaning that a higher dose to the prostatic gland leads to better cancer control (1).

Several randomized trials have shown a benefit of dose escalation to 78–79 Gy for men treated with external radiation for localized PC. Previous data suggested a benefit with even higher doses. In a trial by Coen et al., the safety and efficacy of 82 Gy (a 2 Gy equivalent) delivered with conformal PBT was tested. The estimated rate of ≥Grade 3 late toxicity at 18 months was 6%, indicating that this may be the maximal dose that can be delivered safely with this technique and fractionation (30).

On the other hand, with new techniques such as IMRT (± rapid arc) or IGRT, photon radiation encumbers OAR with low-to-moderate doses. Such doses normally do not cause noticeable side effects for patients, and photon therapy has reached a high level of patient comfort and acceptance. In a similar way, IMRT is described to have excellent efficacy and low toxicity in the treatment of PC, even with elevated final doses (31, 32).

It has, thus, become the standard procedure for RT of PC in many institutes due to the significantly reduced toxicities compared with what has been observed with conventional 3D-approaches.

The feasibility of high-dose IMRT (up to 81 Gy) has been demonstrated in studies with large numbers of patients and has been proven to have comparably low side effects (33–35).

This was stated earlier by Mock et al.: they described IMRT as more effectively enabling dose reductions to OAR in the medium dosage range compared to 3D conformal radiotherapy. Furthermore, they indicated possible benefits of the two-field PBT technique, which reduces doses to surrounding tissues compared to photon-beam RT (36). The physical properties of protons may, thus, decrease common GI and GU side effects even further.

As early as 1983, Duttenhaver et al. discussed proton versus a conventional megavoltage X-Ray (photon) boost, finding no difference in local tumor control (LC), disease-free survival (DFS), or overall survival (OS), yet fewer side effects with an elevated proton boost. Photon RT has evolved since then, as described above, with numerous technical advances, while PBT has yet to prove itself through better LC, OS, or significantly fewer side effects (37).

In dosimetric studies of a small patient group Vargas et al. were able to show a reduced mean rectal (59%) and bladder (35%) dose for PBT compared to IMRT (38). Early outcomes from single arm, prospective trials confirmed these assumptions. Nihei et al. described the incidence of late ≥grade 2 rectal and bladder toxicity at 2 years to be 2.0 and 4.1%, respectively (39).

Similarly, Mendenhall et al. found good early outcomes with image-guided proton therapy, suggesting high efficacy and minimal toxicity with 1.9% grade 3 GU symptoms and <0.5% grade 3 GI toxicities (39, 40). Generally, the dose to healthy tissues in the range <50% of the target prescription was substantially lower with proton therapy (41).

A retrospective analysis of the Medicare database compared early toxicity in 421 men using PBT with 842 matched controls treated with IMRT. A statistically significant decrease in GU toxicity at 6 months for PBT was seen, but this difference had disappeared at one year. There were no other significant differences in toxicity between the two techniques at either 6 or 12 months post-treatment. Yu et al. concluded that although PBT is substantially more cost-intensive than IMRT, no difference in toxicity in a comprehensive cohort of Medicare beneficiaries with PC at 12 months post-treatment was found (42).

Keeping in mind that the amount of bladder exposed to low doses of radiation predicts early toxicity the difference in toxicity seen by Yu et al. is plausible, since in previous studies it has been shown that one improvement in radiation dose distribution for PBT compared to IMRT led to a reduction in the amount of bladder exposed to low and intermediate levels of radiation (36, 41, 43). This dose reduction was most likely responsible for the transient improvement in the Yu study.

However, other studies have found IMRT to be favorable over PBT with regard to toxicity. An analysis from the Medicare Surveillance, Epidemiology, and End Results (SEER) database in the USA identified 684 men treated with PBT between 2002 and 2007 and compared these with a cohort treated with IMRT.

Intensity-modulated radiotherapy was associated with significantly less GI morbidity. However, there were no statistically significant differences in other toxicities, nor a significant difference in the frequency with which patients required additional cancer therapy (44).

There are still no completed randomized trials comparing PBT with photon-beam therapy in men with clinically localized PC (45).

With regard to the OS data, a few major studies have been conducted. One of the major dose-escalation studies was carried out at The Proton Center in Boston. Zietman et al. randomized 393 patients with a PSA <5 ng/ml to a low-dose arm (50.4 Gy photon therapy + 19.8 GyE proton boost) and a high-dose arm (50.4 Gy photon + 28.8 GyE proton boost). The analysis revealed a significant difference in biochemical recurrence-free survival in favor of the high-dose arm. Subgroup analysis of low and high-risk patients (depending on the Gleason score) showed a significant advantage for the high-dose group in both cases. An impact on the OS rate was not observed. Both acute and late toxicities were not increased in either arm compared to the incidence of comparable photon studies (1, 46). However, modern photon treatments allow comparable high-dose application with utmost precision and safety; thus, the latter trial might be termed mainly not as a trial comparing photons and protons, but high-dose to low-dose treatments.

Finally, the American Society for Radiation Oncology (ASTRO) released a list recommending the use of PBT after an evidence-based review for certain tumors, including central nervous system and pediatric malignancies. For others, among them PC, it recommends treatment only within the setting of clinical trials, as there was evidence for the efficacy of PBT but no suggestion that it is superior to photon-based approaches (47, 48).

## Cost Aspect

Concerning costs, several aspects must be highlighted: the construction costs of proton facilities, maintenance costs and outcome, also concerning throughput compared to photon radiation.

First, Keener et al. estimated the building cost for a new PBT center to be between 100 and 250 million US-Dollars. This is the equivalent of about 40 times the price of setting up a state-of-the-art photon radiation center (49).

As for maintenance and cost-effectiveness, Johnstone et al. calculated that a high number (single gantry 85%) of “simple” cases with a faster throughput are necessary for proton facilities to work cost effectively (50).

Proton beam therapy for PC is often described as simple, compared to pediatric or central nervous system indications, where longer setup and/or treatment times are required. A modern proton center requires treating a caseload and emphasizing simple patients with high throughput even before operating costs or any profit are achieved (50). In theory, this means that a PBT facility treating only patients with PC would run cost effectively and profitably.

With regard to compensation, Yu et al. found median Medicare reimbursement in the USA to be over 32,000 US-Dollars for proton RT and over 18,000 for IMRT. In a retrospective study of over 27,000 patients, they found no toxicity difference at 12 months post-treatment, despite the cost being almost double (42).

Furthermore, in a cost utility analysis per quality-adjusted life year (QALY) from a literature search between 2003 and 2013, PBT was not found to be cost-effective in any of the analysis (51).

A very interesting open phase 3 study (NCT01230866) is underway and could make a significant difference to PBT cost-effectiveness, as well as to the life quality of patients. The study compares standard dose of RT (44 treatments) with a hypofractionation concept (five treatments) (52).

This study could dramatically decrease the treatment costs of PBT if the hypofraction arm performs similar or better than normofractionated treatment. It could also improve the life quality of patients as a result of the decreased number of treatment appointments. However, only recently the biological rationale of hypofractionation was revisited questioning the current α/β concepts – newer data assume that prostate α/β are, after all, more closely comparable to those of rectal or other normal tissue than initially believed, thus, questioning the real rationale of hypo-fractionation (53).

In summary, technology for PC RT has made important advances. However, its associated costs have escalated, thus, making cost-effectiveness analysis critical to assess. So far, all aspects of PBT remain far more expensive than photon radiation therapy, meaning that cost consciousness should outweigh standard PBT for PC as long as there is no clear evidence from controlled randomized trials supporting the superiority of PBT, so that the surplus of money spent can be well-invested. However, data are available that proton therapy can be applied, and especially in the pediatric population referral to a high-end particle therapy center should be evaluated. For carbon ions, patients should be treated within clinical trials until the full potential and the biological rationale can be shown in patient treatments.

In conclusion, proton therapy is worth being discussed in modern oncology with assets leading to potentially advantageous treatments; however, this should not lead to unreflected discussions and recommendations that proton therapy is the necessary independently of indication patient age, comorbidities, and other factors. For PC, it will be interesting to follow the ongoing research to see which technique “will be the road less traveled.”

## Conclusion

A high publication rate confirms continued high interest in PBT and its characteristics. Reading through such publications, one gets the impression that the authors often take a similar stance to ASTRO, who finished the abstract of their evidence-based review with the words: “More robust prospective clinical trials are needed to determine the appropriate clinical setting for PBT” (47).

There is much discussion and disagreement concerning toxicities, cost–effectiveness, and the potential for better outcomes (2). However, PBT is certainly cost-intensive and yet has great potential with regard to basic physics and biological principles. Nevertheless, the advantages so far seem to remain theoretical and are brought about by a better dose distribution.

Several trials are underway, among them a multi-institutional randomized phase III Nacional Cancer Institute study (A Phase III Randomized Clinical Trial of Proton Therapy Versus IMRT for low or intermediate risk PC; clinicaltrials.gov ID NCT01617161) comparing PBT to IMRT (54). It is now in its third year and, together with others, will hopefully shed some more light onto the discussion of PC and RT with photons and particles, which in the end will lead to individualized radiotherapy (*i*RT) concepts.

## Conflict of Interest Statement

The authors declare that the research was conducted in the absence of any commercial or financial relationships that could be construed as a potential conflict of interest.

## References

[1] Zietman AL Bae K Slater JD Shipley WU Efstathiou JA Coen JJ Randomized trial comparing conventional-dose with high-dose conformal radiation therapy in early-stage adenocarcinoma of the prostate: long-term results from proton radiation oncology group/american college of radiology 95-09. J Clin Oncol (2010) 28:1106–11. 10.1200/JCO.2009.25.8475 20124169 PMC2834463

[2] Habl G Debus J There is evidence for the superiority of protons and heavy ions, pro radiotherapy in prostate cancer. Med Radiol (2014) 2015:277–89. 10.1007/174_2014_972

[3] U.S. National Institutes of Health (2015). ClinicalTrials.gov

[4] Kosaki K Ecker S Habermehl D Rieken S Jakel O Herfarth K Comparison of intensity modulated radiotherapy (IMRT) with intensity modulated particle therapy (IMPT) using fixed beams or an ion gantry for the treatment of patients with skull base meningiomas. Radiat Oncol (2012) 7:44. 10.1186/1748-717X-7-44 22439607 PMC3338385

[5] Paganetti H Assessment of the risk for developing a second malignancy from scattered and secondary radiation in radiation therapy. Health Phys (2012) 103:652–61. 10.1097/HP.0b013e318261113d 23032895 PMC3464436

[6] Jermann M Particle therapy statistics in 2013. Int J Particle Ther (2014) 1:40–3. 10.14338/IJPT.14-editorial-2.1

[7] Combs SE Schulz-Ertner D Herfarth KK Krempien R Debus J. [Advances in radio-oncology. From precision radiotherapy with photons to ion therapy with protons and carbon ions]. Chirurg (2006) 77:1126–32. 10.1007/s00104-006-1268-2 17119885

[8] Niederhuber JE Armitage JO Doroshow JH Kastan MB Tepper JE Abeloff’s Clinical Oncology. Philadelphia: Elsevier (2014).

[9] Kanai T Matsufuji N Miyamoto T Mizoe J Kamada T Tsuji H Examination of GyE system for HIMAC carbon therapy. Int J Radiat Oncol Biol Phys (2006) 64:650–6. 10.1016/j.ijrobp.2005.09.043 16414376

[10] DeLaney TF. Proton therapy in the clinic. Front Radiat Ther Oncol (2011) 43:465–85. 10.1159/000322511 21625169

[11] Delaney TF Kooy HM Protons and Charge Particle Radiotherapy. Philadelphia: Lippincott Williams & Wilkins (2008).

[12] Egger E Zografos L Schalenbourg A Beati D Bohringer T Chamot L Eye retention after proton beam radiotherapy for uveal melanoma. Int J Radiat Oncol Biol Phys (2003) 55:867–80. 10.1016/S0360-3016(02)04200-1 12605964

[13] Miralbell R Lomax A Cella L Schneider U. Potential reduction of the incidence of radiation-induced second cancers by using proton beams in the treatment of pediatric tumors. Int J Radiat Oncol Biol Phys (2002) 54:824–9. 10.1016/S0360-3016(02)02982-6 12377335

[14] Munzenrider JE. Proton therapy for uveal melanomas and other eye lesions. Strahlenther Onkol (1999) 175(Suppl 2):68–73. 10.1007/BF03038893 10394402

[15] Brenner DJ Hall EJ. Secondary neutrons in clinical proton radiotherapy: a charged issue. Radiother Oncol (2008) 86:165–70. 10.1016/j.radonc.2007.12.003 18192046

[16] Chung CS Yock TI Nelson K Xu Y Keating NL Tarbell NJ. Incidence of second malignancies among patients treated with proton versus photon radiation. Int J Radiat Oncol Biol Phys (2013) 87:46–52. 10.1016/j.ijrobp.2013.04.030 23778197

[17] Paganetti H. Range uncertainties in proton therapy and the role of Monte Carlo simulations. Phys Med Biol (2012) 57:R99–117. 10.1088/0031-9155/57/11/R99 22571913 PMC3374500

[18] Yoon M Shin D Kwak J Park S Lim YK Kim D Characteristics of movement-induced dose reduction in target volume: a comparison between photon and proton beam treatment. Med Dosim (2009) 34:191–201. 10.1016/j.meddos.2008.08.004 19647628

[19] Frey K Unholtz D Bauer J Debus J Min CH Bortfeld T Automation and uncertainty analysis of a method for in-vivo range verification in particle therapy. Phys Med Biol (2014) 59:5903–19. 10.1088/0031-9155/59/19/5903 25211629 PMC10008084

[20] Mackin D Peterson S Beddar S Polf J. Evaluation of a stochastic reconstruction algorithm for use in Compton camera imaging and beam range verification from secondary gamma emission during proton therapy. Phys Med Biol (2012) 57:3537–53. 10.1088/0031-9155/57/11/3537 22588144 PMC3392092

[21] Habermehl D Henkner K Ecker S Jakel O Debus J Combs SE. Evaluation of different fiducial markers for image-guided radiotherapy and particle therapy. J Radiat Res (2013) 54(Suppl 1):i61–8. 10.1093/jrr/rrt071 23824129 PMC3700523

[22] Habermehl D Naumann P Bendl R Oelfke U Nill S Debus J Evaluation of inter- and intrafractional motion of liver tumors using interstitial markers and implantable electromagnetic radiotransmitters in the context of image-guided radiotherapy (IGRT) – the ESMERALDA trial. Radiat Oncol (2015) 10:143 10.1186/s13014-015-0456-y 26169281 PMC4499938

[23] Richter D Graeff C Jakel O Combs SE Durante M Bert C. Residual motion mitigation in scanned carbon ion beam therapy of liver tumors using enlarged pencil beam overlap. Radiother Oncol (2014) 113:290–5. 10.1016/j.radonc.2014.11.020 25465732

[24] Shioyama Y Tsuji H Suefuji H Sinoto M Matsunobu A Toyama S Particle radiotherapy for prostate cancer. Int J Urol (2015) 22:33–9. 10.1111/iju.12640 25308767

[25] Tsujii H Kamada T. A review of update clinical results of carbon ion radiotherapy. Jpn J Clin Oncol (2012) 42:670–85. 10.1093/jjco/hys104 22798685 PMC3405871

[26] Chen GT Castro JR Quivey JM Heavy charged particle radiotherapy. Annu Rev Biophys Bioeng (1981) 10:499–529. 10.1146/annurev.bb.10.060181.002435 7020583

[27] Ishikawa H Tsuji H Kamada T Akakura K Suzuki H Shimazaki J Carbon-ion radiation therapy for prostate cancer. Int J Urol (2012) 19:296–305. 10.1111/j.1442-2042.2012.02961.x 22320843

[28] Nomiya T Tsuji H Maruyama K Toyama S Suzuki H Akakura K Phase I/II trial of definitive carbon ion radiotherapy for prostate cancer: evaluation of shortening of treatment period to 3 weeks. Br J Cancer (2014) 110:2389–95. 10.1038/bjc.2014.191 24722181 PMC4021525

[29] Zhu XR Poenisch F Li H Zhang X Sahoo N Wu RY A single-field integrated boost treatment planning technique for spot scanning proton therapy. Radiat Oncol (2014) 9:202. 10.1186/1748-717X-9-202 25212571 PMC4262206

[30] Coen JJ Bae K Zietman AL Patel B Shipley WU Slater JD Acute and late toxicity after dose escalation to 82 GyE using conformal proton radiation for localized prostate cancer: initial report of American College of Radiology Phase II study 03-12. Int J Radiat Oncol Biol Phys (2011) 81:1005–9. 10.1016/j.ijrobp.2010.06.047 20932675

[31] Cahlon O Hunt M Zelefsky MJ. Intensity-modulated radiation therapy: supportive data for prostate cancer. Semin Radiat Oncol (2008) 18:48–57. 10.1016/j.semradonc.2007.09.007 18082588

[32] Cahlon O Zelefsky MJ Shippy A Chan H Fuks Z Yamada Y Ultra-high dose (86.4 Gy) IMRT for localized prostate cancer: toxicity and biochemical outcomes. Int J Radiat Oncol Biol Phys (2008) 71:330–7. 10.1016/j.ijrobp.2007.10.004 18164858

[33] Zelefsky MJ Chan H Hunt M Yamada Y Shippy AM Amols H. Long-term outcome of high dose intensity modulated radiation therapy for patients with clinically localized prostate cancer. J Urol (2006) 176:1415–9. 10.1016/j.juro.2006.06.002 16952647

[34] Zelefsky MJ Fuks Z Hunt M Lee HJ Lombardi D Ling CC High dose radiation delivered by intensity modulated conformal radiotherapy improves the outcome of localized prostate cancer. J Urol (2001) 166:876–81. 10.1097/00005392-200109000-00017 11490237

[35] Zelefsky MJ Fuks Z Hunt M Yamada Y Marion C Ling CC High-dose intensity modulated radiation therapy for prostate cancer: early toxicity and biochemical outcome in 772 patients. Int J Radiat Oncol Biol Phys (2002) 53:1111–6. 10.1016/S0360-3016(02)02857-2 12128109

[36] Mock U Bogner J Georg D Auberger T Potter R. Comparative treatment planning on localized prostate carcinoma conformal photon- versus proton-based radiotherapy. Strahlenther Onkol (2005) 181:448–55. 10.1007/s00066-005-1317-7 15995838

[37] Duttenhaver JR Shipley WU Perrone T Verhey LJ Goitein M Munzenrider JE Protons or megavoltage X-rays as boost therapy for patients irradiated for localized prostatic carcinoma. An early phase I/II comparison. Cancer (1983) 51:1599–604. 10.1002/1097-0142(19830501)51:9<1599::AID-CNCR2820510908>3.0.CO;2-O 6299503

[38] Vargas C Fryer A Mahajan C Indelicato D Horne D Chellini A Dose-volume comparison of proton therapy and intensity-modulated radiotherapy for prostate cancer. Int J Radiat Oncol Biol Phys (2008) 70:744–51. 10.1016/j.ijrobp.2007.07.2335 17904306

[39] Nihei K Ogino T Onozawa M Murayama S Fuji H Murakami M Multi-institutional Phase II study of proton beam therapy for organ-confined prostate cancer focusing on the incidence of late rectal toxicities. Int J Radiat Oncol Biol Phys (2011) 81:390–6. 10.1016/j.ijrobp.2010.05.027 20832180

[40] Mendenhall NP Li Z Hoppe BS Marcus RB Jr Mendenhall WM Nichols RC Early outcomes from three prospective trials of image-guided proton therapy for prostate cancer. Int J Radiat Oncol Biol Phys (2012) 82:213–21. 10.1016/j.ijrobp.2010.09.024 21093164

[41] Trofimov A Nguyen PL Coen JJ Doppke KP Schneider RJ Adams JA Radiotherapy treatment of early-stage prostate cancer with IMRT and protons: a treatment planning comparison. Int J Radiat Oncol Biol Phys (2007) 69:444–53. 10.1016/j.ijrobp.2007.03.018 17513063 PMC2695934

[42] Yu JB Soulos PR Herrin J Cramer LD Potosky AL Roberts KB Proton versus intensity-modulated radiotherapy for prostate cancer: patterns of care and early toxicity. J Natl Cancer Inst (2013) 105:25–32. 10.1093/jnci/djs463 23243199 PMC3536640

[43] Karlsdottir A Johannessen DC Muren LP Wentzel-Larsen T Dahl O. Acute morbidity related to treatment volume during 3D-conformal radiation therapy for prostate cancer. Radiother Oncol (2004) 71:43–53. 10.1016/j.radonc.2004.01.014 15066295

[44] Sheets NC Goldin GH Meyer AM Wu Y Chang Y Sturmer T Intensity-modulated radiation therapy, proton therapy, or conformal radiation therapy and morbidity and disease control in localized prostate cancer. JAMA (2012) 307:1611–20. 10.1001/jama.2012.460 22511689 PMC3702170

[45] DiBiase SJ External Beam Radiation Therapy for Localized Prostate Cancer (2015). Available from: http://www.uptodate.com

[46] Zietman AL DeSilvio ML Slater JD Rossi CJ Jr Miller DW Adams JA Comparison of conventional-dose vs high-dose conformal radiation therapy in clinically localized adenocarcinoma of the prostate: a randomized controlled trial. JAMA (2005) 294:1233–9. 10.1001/jama.294.10.1233 16160131

[47] Allen AM Pawlicki T Dong L Fourkal E Buyyounouski M Cengel K An evidence based review of proton beam therapy: the report of ASTRO’s emerging technology committee. Radiother Oncol (2012) 103:8–11. 10.1016/j.radonc.2012.02.001 22405807

[48] Nguyen PL Trofimov A Zietman AL. Proton-beam vs intensity-modulated radiation therapy. Which is best for treating prostate cancer? Oncology (2008) 22:748–54; discussion 754, 757. 18619120

[49] Keener AB Arrests reveal debate about costs and benefits of proton therapy. Nat Med (2014) 20:1081 10.1038/nm1014-1081 25295925

[50] Johnstone PA Kerstiens J Richard H. Proton facility economics: the importance of “simple” treatments. J Am Coll Radiol (2012) 9:560–3. 10.1016/j.jacr.2012.03.014 22863464

[51] Amin NP Sher DJ Konski AA. Systematic review of the cost effectiveness of radiation therapy for prostate cancer from 2003 to 2013. Appl Health Econ Health Policy (2014) 12:391–408. 10.1007/s40258-014-0106-9 25022451

[52] Study of Hypo-Fractionated Proton Radiation for Low Risk Prostate Cancer. ClinicalTrials.gov, NCT01230866. Proton Collaborative Group (2015).

[53] Baumann M Holscher T Denham J Fractionation in prostate cancer – is it time after all? Radiother Oncol (2010) 96:1–5. 10.1016/j.radonc.2010.06.001 20566227

[54] Proton Therapy vs. IMRT for Low or Intermediate Risk Prostate Cancer (PARTIQoL). ClinicalTrials.gov, NCT01617161.

